# Primary Healthcare of black immigrants during the COVID-19 pandemic^*^

**DOI:** 10.1590/1980-220X-REEUSP-2022-0441en

**Published:** 2023-09-15

**Authors:** Flaviane Andreele Jacinto da Silva, Aida Maris Peres, Rafaela Gessner Lourenço, Marli Aparecida Rocha de Souza, Karla Crozeta Figueiredo, Climene Laura de Camargo

**Affiliations:** 1 Universidade Federal do Paraná, Programa de Pós-Graduação em Enfermagem, Curitiba, PR, Brazil.; 2 Universidade Federal da Bahia, Escola de Enfermagem, Departamento de Enfermagem, Salvador, BA, Brazil.

**Keywords:** Primary Healthcare, Health of Ethnic Minorities, Health Vulnerability, Racism, Atención Primaria de Salud, Salud de las Minorías Étnicas, Vulnerabilidad en Salud, Racismo, Atenção Primária à Saúde, Saúde das Minorias Étnicas, Vulnerabilidade em Saúde, Racismo

## Abstract

**Objective:**

To analyze how the healthcare of black immigrants was conducted during the COVID-19 pandemic in Primary Healthcare.

**Method:**

An exploratory-descriptive study with a qualitative approach, carried out through semi-structured interviews with professionals who worked in 10 Health Units in the city of Curitiba, Brazil, from October 2020 to January 2021. Structural racism was the conceptual framework. The statements were submitted to content analysis after using the MAXQDA program as support.

**Results:**

A total of 21 professionals from the multidisciplinary team participated and three categories emerged from the analyzes: Healthcare for black immigrants in PHC during the Covid-19 pandemic; Limits and potentialities of PHC for healthcare for black immigrants; Structural racism in PHC practices aimed at black immigrants.

**Conclusion:**

Action in the pandemic was guided by protocols that did not expand healthcare to vulnerable populations, including black immigrants. The main barrier was communication, as most black immigrants in the surveyed locations were Haitians. Structural racism was identified in professional practice.

## RESUMO

**Objetivo:**

Analisar como foi realizada a atenção à saúde do imigrante negro durante a pandemia da covid-19 na Atenção Primária à Saúde.

**Método:**

Pesquisa exploratória-descritiva de abordagem qualitativa, realizada por meio de entrevistas semiestruturadas, com profissionais que atuavam em dez Unidades de Saúde do município de Curitiba, de outubro de 2020 a janeiro de 2021. O racismo estrutural constituiu o referencial conceitual. Os depoimentos foram submetidos à análise de conteúdo após utilização do *software* MAXQDA como suporte.

**Resultados:**

Participaram 21 profissionais da equipe multiprofissional e das análises emergiram três categorias: atenção à saúde do imigrante negro na APS durante a pandemia da covid-19; limites e potencialidades da APS para atenção à saúde do imigrante negro; racismo estrutural nas práticas da APS voltadas ao imigrante negro.

**Conclusão:**

A atuação na pandemia foi pautada por protocolos que não ampliaram a atenção à saúde às populações vulneráveis, entre elas, a de imigrantes negros. A principal barreira foi a comunicação, pois a maioria dos imigrantes negros nos locais pesquisados eram haitianos. O racismo estrutural foi identificado na prática profissional.

## RESUMEN

**Objetivo:**

Analizar la ejecución de la atención sanitaria del inmigrante negro durante la pandemia de COVID-19 en la Atención Primaria

**Método:**

Es una investigación exploratoria-descriptiva de abordaje cualitativo, realizada a profesionales que actúan en 10 Unidades de Salud de la ciudad de Curitiba a través de entrevistas semiestructuradas, entre octubre de 2020 y enero de 2021. El racismo estructural determinó el marco conceptual. Los testimonios se sometieron a análisis de contenido después de utilizar como soporte el software MAXQDA.

**Resultados:**

Participaron 21 profesionales del equipo multidisciplinario y de los análisis surgieron tres categorías: Atención a la salud del inmigrante negro en la APS durante la pandemia de Covid-19; Límites y potencialidades de la APS para la atención sanitaria del inmigrante negro; Racismo estructural en las prácticas de APS dirigidas al inmigrante negro.

**Conclusión:**

La actuación en la pandemia estuvo basada en protocolos que no expandieron la atención sanitaria a las poblaciones vulnerables, entre ellas, la del inmigrante negro. La principal barrera era la comunicación, ya que la mayoría de los inmigrantes negros de los lugares estudiados eran haitianos. Se identificó racismo estructural en la práctica profesional.

## INTRODUCTION

According to the World Health Organization (WHO), there are currently around 1 billion immigrants^(1)^. Migrant is a comprehensive term by definition, as it does not stipulate whether that individual migrated within their own national territory or across country borders. The term immigrant characterizes those who migrate or cross the border of a country, making this new geographic space their current residence^(2)^.

Some countries stand out among those that receive the most immigrants. Among them: Canada, a country that facilitates the entry of foreigners, focusing on younger people, fluent in the native languages and qualified; the United States, which opts for qualified immigrants, especially in the most needy areas of the country; and Brazil, which is among the countries which receive refugee immigrants characterized by coming from countries with economic issues that aggravate the social condition of the population, leading many to migrate in search of opportunities, legally or not^(3)^.

In this context, the demand for Brazil as an option for immigrants has grown in recent years. A total of 774,200 immigrants were registered in the country in the period from 2011 to 2018. In all, 492,700 had a long-term record, meaning they had stayed in the country for more than one year. The most registered nationalities of immigrants in Brazil in 2018 were: Venezuelans (39%), Haitians (14.7%), Colombians (7.7%), Bolivians (6.8%) and Uruguayans (6.7%)^(4)^.

It is known that immigrants represent a portion of the population exposed to different difficulties in periods of financial and social crisis, especially social and health. In the context of the Covid-19 pandemic, the unemployment rate rose and generated underemployment conditions, and in the same proportion intensified the vulnerability of the immigrant population, which worsened their economic situation, resulting in the loss of livelihood conditions^(5)^. The term vulnerability presented in this study was used with the concept of guaranteeing the rights of politically fragile populations^(6)^.

The right of immigrants to health services in Brazil is guaranteed by the Federal Constitution of 1988, which provides that every individual in the national territory can access health services. However, evidence in the literature indicates that healthcare for the black population has obstacles, including the unpreparedness of professionals to promote comprehensiveness, a fact which can be seen in the face of structural racism and is capable of generating inequality in this care^(7)^.

In addition, it is known that the Covid-19 pandemic has greatly affected the black population in Brazil. Data released by the Ministry of Health (*Ministério da Saúde - MS*) until May 2020 indicate that 54.8% of the 8,963 black patients hospitalized in Rio de Janeiro died in hospitals, while the lethality rate among white patients (n = 9,998) was 37.9%^(8)^. However, there is a gap in information about the immigrant’s condition in this context, since official data from the Hospital Information System (*Sistema de Informações Hospitalares - SIH*) and the Covid-19 disease notification do not present records about the color and ethnicity of the user^(5)^.

Therefore, it is important to highlight the need to problematize racism in its various existing nuances, since it can influence the healthcare process of black immigrants in Brazil. Structural racism is defined as an exposed situation that conditions its structures with the frequent maintenance of the race element, which relegates the black body to subordinate conditions. This element constitutes a structuring element in social power agencies, whether by structural violence manifested in the absence of rights, in cultural violence presupposed by incapacity or incivility, or by institutional strength^(9)^.

The concept of structural racism is inherent to the social order and goes beyond the space of individual action, reaching the institutional dimension through imposing rules and standards as a result of the regularity that constitutes and determines political, economic, legal and family relationships. Structural racism goes beyond individual conditions or institutional processes, as it constitutes a rule and not an exception, as it interferes in the daily lives of the black population and manifests itself in the spaces that permeate the daily life of the black immigrant^(9)^.

Considering the fact that a significant part of the contingent of immigrants who access the Brazilian territory declare themselves brown or black according to the classification system by color or race of the Brazilian Institute of Geography and Statistics (*Instituto Brasileiro de Geografia e Estatística - IBGE*), this study is supported by the concept of structural racism, and aimed to analyze how the healthcare of black immigrants was conducted during the COVID-19 pandemic in Primary Healthcare (PHC).

## METHOD

### Study Design

This is an exploratory and descriptive study with a qualitative approach. The data source comprised interviews with professionals who work in PHC in the city of Curitiba, PR, Brazil.

### Location, Population and Selection Criteria

We initially started with a survey of three Non-Governmental Organizations (NGOs) that serve the black immigrant population in the municipality of the study to select the Health Units (HUs) which composed the research scenarios. This enabled mapping the four regions with higher concentrations of this population. Based on this survey, the study was carried out in 10 HUs distributed in four of the 10 health districts that compose the municipality, located in the west, south and central regions. Thus, it was possible to locate the HU that registered the largest enrolled population characterized as black immigrants. The following inclusion criteria were defined: professionals working in healthcare at the selected HU. Exclusion criteria were not defined. The professionals were invited to participate in the study at the place of work and there were no refusals to participate.

### Data Collection

The semi-structured instrument for data collection was developed by the researchers and consisted of six questions that dealt with how healthcare was provided to black immigrants in the HU during the Covid-19 pandemic. In addition, the instrument made it possible to identify how black immigrants arrived at the HU or how they were recognized. The interviews were conducted in person by one of the authors, between October 2020 and January 2021, with an average duration of 10min’30”, with a maximum variation of 15min’49” and a minimum of 4min’48”. The interviews were audio-recorded and fully transcribed using the Word text editor, and the transcripts were not returned for comments by the participants.

### Data Analysis and Treatment

Data analysis took place using the content analysis technique in the thematic modality^(10)^, consisting of pre-analysis, material exploration, treatment of results, interpretation and inference steps. The MAXQDA program was used as support for data analysis. This tool is an operating system which assists in analyzing qualitative data using different documents with various formats, texts, images, focus groups, audio and videos, with the aim of assisting the researcher in the analysis. The choice of this program is justified due to its innovative nature and because it demonstrates rigorous criteria for data organization in qualitative research. The Consolidated criteria for reporting qualitative research (COREQ) was used to check the stages of the method in this study.

### Ethical Aspects

The study was approved by the Research Ethics Committee of the Health Sciences Sector of the Federal University of Paraná (UFPR) under number 4,216,553 and in the Municipal Health Secretariat (*Secretaria Municipal de Saúde - SMS*) under number 4,251,498 in 2020. It also followed all precepts of Resolution no. 466/2012, with the signing of the Informed Consent Form (ICF) for all participants before each interview. An abbreviation of the interviewee’s professional category was adopted to identify the participants’ speeches and maintain anonymity: community agent (CA); nursing assistant (NA); assistant/technician in oral health (AO); nursing technician (NT); dentist (DE); nurse (N) and doctor (DOC), followed by Arabic numerals.

## RESULTS

The study had 21 participants from the professional categories presented below, with the respective average time of work in the current health unit: three CAs, with an average performance of 17 years and five months; two NAs with an average duration of 12 years and five months; two AOs, with an average of 17 years and five months; three NTs, with an average performance of 11 years and five months; two DEs, with an average performance of 14 years and nine months; five Ns, with an average performance of six years and five months; and four DOCs, with an average performance of six years and one month.

**Figure 1:**
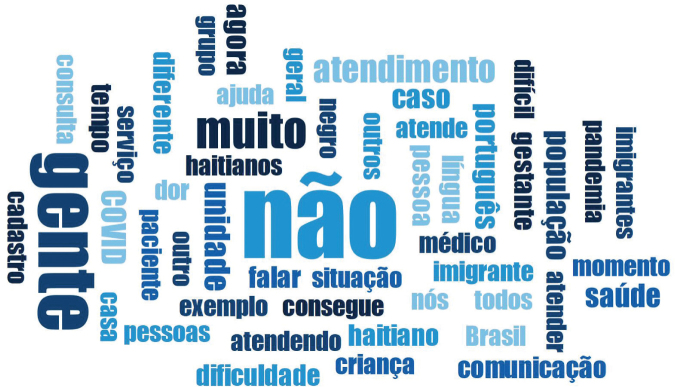
Word cloud produced from participants' speech by the MAXQDA program. Curitiba, PR, Brazil, 2021.

Source: The authors.

A word cloud which stood out in the participants’ speeches was built using the MAXQDA program, which enabled a semantic reading of the central questions issued. The cloud was formed after removing connecting verbs and common words, such as: the, that, one, of, thus, soon. Then, the five most frequent words were selected: no (447), people (257), a lot (112), service (58), unit (47). The shape of the cloud can be seen in Figure 1.

The term “no” among the words which stood out the most was commonly used to refer to the issue of non-differentiation in care provided to black immigrants, the condition and difficulty of communication; the term “people” was used in a context of reference to the multidisciplinary team; the term “very” was reported to express appreciation/exacerbation of some characteristic of black immigrants; the term “service” appeared in the indication that the healthcare offered is the same for any user, as predicted by the care flow during the Covid-19 pandemic; and the term “unit” referred to the HU investigated at the time of the interview.

Next, three empirical categories emerged from the content analysis of the interviewees’ reports, defined *a posteriori*: Healthcare for black immigrants in PHC during the Covid-19 pandemic; Limits and potentialities of PHC for healthcare for black immigrants; and Structural racism in PHC practices aimed at black immigrants.

### Healthcare for Black Immigrants in PHC During the Covid-19 Pandemic

In the context of healthcare for black immigrants, professionals highlighted the conditions of social vulnerability with which black immigrants are identified in the service, especially with regard to housing, employment and food:

*I had one who came, a Haitian couple, who came from Haiti and they came here with the difficulties that they arrived with (...) without a job, without anything, so they came here to be assisted (...) (CA 01)*

In addition, professionals reported how this population accesses the investigated services. They highlighted that the search is direct for the service, but that the indication about the location and the right to use the HU in the territory is usually carried out spontaneously among the immigrant community, those who have resided in Brazil longer, for the newcomers:

*(...) what I know is that they come a lot by direct search, so people know that there is a prenatal service and as they are already pregnant, they end up coming. (DOC 02)*

*The unit? I think they arrive through others (...) for example, they (...) one comes, already brings another colleague through information between them there, you know? They like the service, so they tell someone else and that’s how they communicate with each other. They register, the community agent goes to the house. (AO 01)*

The documentation provided by immigrants to ensure regular access to the health service is the passport, which proves their status as a foreigner. In addition, the guarantee of equal care by immigrants who seek the service was highlighted:

*They end up bringing the passport, we end up registering them and we link it. (CA 02)*

*So the conduct is the same, they have the same rights as Brazilians, they will go through consultation, follow-up and everything else. (NT 03)*

In addition, the following stand out regarding the main health needs of the black immigrant population identified by the participants: respiratory symptoms, gynecological/obstetric, pediatric, sexually transmitted infections, and chronic diseases, such as diabetes and hypertension:

*The most common cases that appear here are either respiratory symptomatic, or Haitian pregnant women, it would be more gynecology and pediatrics. (N 02)*

*(...) they put on a lot of weight. Then sometimes they have hypertension, we got some diabetics, a device is provided, we monitor it, they come, they do BP control (Blood Pressure) (...) everything. (N 01)*

The participants reported that there was no specific organization in the care of immigrants, including black immigrants during the Covid-19 pandemic, and the organizational logistics focused on isolating users with respiratory symptoms in a certain physical space of the HU and those with general symptoms in another. It should be noted that one of the HU did not provide care for symptomatic users of Covid-19, as it began to care for gynecological and pediatric symptoms with the restructuring of services. The focus in this service was to organize a physical space for pregnant women in a safer way, as they represent a vulnerable group to the disease development.

*As we are not directly assisting Covid, our unit is providing more vaccinations, ultrasound, attending specialized cases. As for the basics, it would be more gynecology and pediatrics. She came for gynecology, only the Covid part is going there for (name of another unit). (NA 03)*

*Specific? No, because the service was on the case, it wasn’t specific for immigrants and everything else. In this case, the health unit was prepared to serve the general population. (NT 03)*

It was noteworthy that one of the interviewees highlighted their perception of the non-protagonism of PHC in facing the Covid-19 pandemic in the investigated municipality:

*(...) unfortunately, in the triad between care, management and epidemiology, the epidemic ended up leading the actions. (...) after all, it was in the middle of a pandemic, but primary care was not the protagonist of the actions, so the health units did it their own way. (N 02)*

It is observed that only one of the interviewees mentioned the National Policy for the Comprehensive Health of the Black Population (*Política Nacional de Saúde Integral da População Negra*) as ordering care for the black immigrant population in PHC. In addition, the interviewees explained that they did not receive any type of training or specific material for this population group, leaving the professional to seek training and improvements on their own initiative, motivated by personal interest in the subject.

*I think they (immigrants) would fit into the Health Care Policy for the Black Population (...) (N 05)*

*None, on the part of the governments, there was nothing. There are groups that I am engaged with, so I had training in an autism group, I had training in women’s defense groups, you know, I had lectures by the black conscience movement, I had a lecture by the Colombian consulate, but all online and all by looking for mine. Yes, it was individual.. (NT 02)*

### Limits and Potentialities of PHC for Healthcare for Black Immigrants

The linguistic barrier was highlighted regarding the limits identified for the healthcare of black immigrants, since many of them do not speak Portuguese, for example the Haitians. It is noteworthy that the participants did not point out this communication difficulty when referring to immigrants from Portugal, Mozambique and Angola, as these are Portuguese-speaking countries.

*Yeah, now I remember, it’s a bit difficult for some immigrants who don’t understand Portuguese. (N 04)*

*Challenging (...)the issue (...) is that they don’t speak Portuguese, most speak Creole or speak French, and we are not specialized in these languages. So, the difficulty is (...) communication (...) even with them, you know? (...) (NA 01)*

Another limiting element identified was the cultural differences that black immigrants have. Sometimes, these differences were understood by professionals as triggers of habits that can influence prevention and health promotion actions.

*We have a lot of sayings of our own. For someone from another country, there are traditions and customs, it’s their culture, so it’s very different, right (...), but I don’t know what they do? We don’t know how to approach traditions and customs in a way that doesn’t, that doesn’t affect the black population, because it seems that here they live in micro feuds, they value their language a lot, they speak their language, their food is adapted with what they have here, so they value it a lot. Trying to show them how prevention and health promotion can be done is a challenge. (N 05)*

Community unity was highlighted with regard to potentialities in providing care for the black immigrant population. For example, a black immigrant who is fluent in Portuguese can serve as an interpreter for someone who does not speak the language during a consultation as a strategy to overcome the communication barrier.

*(...), but they have rights to the SUS, to equal care at the unit. Sometimes what helps is when a friend comes along, someone we know, someone who can talk a little more that helps us understand, the bond they have with each other. (NA 03)*

The participants pointed out that the HU represents a welcoming place, which highlights the importance of creating a bond with the population for the effectiveness of healthcare.

*I think we are their mother house, they need us, they often not only seek the clinical part, but we realize that it is more even the psychological part, we are the mother house for them. Yes, they come for help. (...) with some we create a bond, you know, we manage to maintain a bond. And one of our skills is welcoming, as we welcome them very well (...) (N 01)*

Other discourses pointed to some characteristics of this group as a positive aspect in caring for the black immigrant population, such as joy, good humor and kindness. A speech that drew attention was the perception that Brazil is a country that welcomes immigrants.

*(...) but they’re happy people too, they don’t come like that, they’re not like the other users we have, they’re happier when you end up winning them over, right. (N 03)*

*I think they are very kind, they welcome us in their house, you know, they come with the notebook, they show me everything that is at home. They are very kind (...) I think that Brazil welcomes immigrants. Now that doesn’t happen out there. (CA 02)*

Some strategies were listed to qualify healthcare for black immigrants in PHC. Among them, the use of applications with simultaneous translation. Another strategy was to exchange instant messages through applications, for example, WhatsApp, as a tool to improve communication and group creation with these users. Also mentioned was the development of a dictionary with the most used words in the languages of Haitian immigrants and the translation of the pregnant woman’s card into the Creole and French dialects.

*(...) then the doctor uses the cell phone translator, sometimes there is a resident who even speaks the language more or less, helps to translate a little, but it is more complicated (...) to assist them. (NA 01)*

*And then, we were setting up a group, I even have Haitians here (...) so I was doing (...) Ahem, a WhatsApp group just for Haitians so we can talk to them. (CA 01)*

*Also a translation of the card (...) and also a glossary (...) there was a list of words (...) a dictionary (...) a dictionary of some words? That, of some words most used by us and by them. Okay, then it helps, right? (DE 01)*

### Structural Racism in PHC Practices Aimed at Black Immigrants

The interviews revealed the structural racism veiled in the professionals’ speech, as in the passages in which there is a negative manifestation about the need for attention directed to the racial issue, with the justification that people have the same rights and therefore are treated equally in the services.

*We didn’t see them as black or immigrants, they are people, you know? (...) it’s hard for us to be talking like that, because we serve them the same as everyone else, (...), for me it doesn’t matter if he’s white, black, yellow (...) (N 01)*

*I can’t tell you, I don’t know, because they end up being approached like all other users, right, in fact there is no discrimination (...) the treatment or special care for them, Venezuelan, Haitian is the same, right, it’s the same right that Brazilians have. (NT 03)*

The speech of one of the participants stood out for recognizing structural racism among professionals, pointing out that some see all immigrants as Haitians, as the service also serves black immigrants from other countries, such as Angola and Mozambique.

*(...) I even find it funny that they call everyone who is black a Haitian immigrant. So, sometimes the person is African, but speaks Portuguese, comes from Angola, comes from Mozambique and such (...) (DOC 02)*

Another fact that points to the existence of veiled structural racism in the participants’ discourse is the perception of their hygiene habits, which is understood as something positive and even surprising.

*(...) this is a very interesting thing that they are very careful with their health (...) sometimes the babies come all dressed up like that, the mothers are very careful (...) they come very clean, very well dressed, very well taken care of, we notice that they care a lot (...) (DE 01)*

Two speeches pointed to the perception of the existing gender determination in the affective relationships of black immigrants, with emphasis on female subjugation.

*Today men have a greater command of the language, women still do not and men are always present during the assistance of their companions. Sometimes it is as an interpreter, sometimes it seems like a relationship with a different profile than what we have here in our country (...) (N 02)*

*(...) I see mainly when they are accompanied by their partners, by their husbands, they come with a lower tolerance to nausea, pain. We realize that, when they are alone, they are stronger, so there is a different relationship when they are with a partner, you know? They victimize themselves more, perhaps as a way to call attention. (DOC 01)*

Veiled structural racism was also identified in speeches that generalized the behavior of this population as immediatist, and of victimization due to the vulnerable condition they carry, as a way of guaranteeing advantage or priority in healthcare.

*(...) there are situations in which we realize that one can wait a little bit, that even though she is a bit anguished wanting to solve her problem, but I have someone in the queue right behind her, I am seeing a person who needs more than her at that moment, and not only that, I’ve had situations like this, with black people, with gay people, with HIV, so, everyone who represents a minority, when they see that they are not going to get care the way they want it at that moment, of course not all, I’m talking about some, some representatives, so they end up using the minority they represent, as if we were discriminating. (NT 02)*

*There are some Haitians that I think are like that, they are very immediate, they arrive, they want to be seen right away, and if they have (...) they sometimes don’t want to wait (...) (CA 01)*

## DISCUSSION

The professional category with the longest experience in the HU investigated was that of Community Health Agents (CHA), who constitute fundamental professionals in healthcare as they are the link between the population and the health team. This context is fundamental with regard to the healthcare of immigrants, as it strengthens the bond between the health team and favors the search for care^(11)^.

The difficulty in communicating with immigrants was highlighted in the word cloud, being one of the main confrontations that health professionals portrayed in providing healthcare for the black immigrant population. Without effective communication, there is a barrier for the user to understand their health status, prescription of medication or care. Thus, in addition to not understanding the referral flows, errors may occur in the dosage of medications, for example, but mainly, the difficulty in creating a bond with the user^(12)^.

The access barrier due to language difficulties is highlighted in the literature as a potential impediment to expressing the needs of immigrants and providing guidance by professionals. However, the social inclusion of this population must be understood as a recognized right in Brazil^(13)^. Thus, the impact on establishing a bond caused by the language barrier is worrying, as it occurs in healthcare and is essential to approach and build a bond of trust between the user and the healthcare team. In addition, this limitation may interrupt treatment continuity, and prevent the professional from promoting effective and resolving actions^(14)^.

As a way to face this problem, the participants of this study cited the use of communication tools, including information technologies. In the approach to using the WhatsApp tool in a HU, it was identified that in addition to it being characterized as a communication channel, it has also become a strategy for carrying out health education actions, especially in groups that bring together immigrants from different nationalities. Therefore, it is considered that in addition to facilitating the user’s approach to the service by enabling sharing and support, the tool can also be considered an alternative to intervene in the health and disease process, highlighted in the context of health during the pandemic period by its pertinence and expansion of care^(15)^.

Another strategy pointed out was the translation of the pregnant woman’s card, which was performed in partnership between a public university and the Municipal Health Department into Creole and French. This initiative sought to break down barriers in healthcare so that immigrant women could better understand their gestational process and was characterized as a facilitator in communication^(16)^. The construction of instruments and tools such as glossaries and dictionaries which aim to ensure communication between health professionals and the immigrant population is opportune. In doing so, there is the prerogative of more assertive care in prevention and health promotion^(14)^.

Initiatives like these are in line with the precepts that guide the National Policy for the Comprehensive Health of the Black Population, although this Policy was rarely mentioned by the participants. It should be noted that its objective is to promote equity in achieving the human right to health, aiming to guarantee comprehensive care and promotion of racial equality in health, as well as to correct the inequities resulting from the historical discrimination experienced by the black population. These facts are little discussed in the literature, which points to a lack of visibility of this Policy and the development of other specific materials aimed at the healthcare of this population. This situation determines that training to care for vulnerable populations is often linked to an individual initiative and motivated by the personal desires of professionals^(8)^.

Immigrants to Brazil are welcomed in the PHC with the guarantee of access to health for everyone in the national territory. This may influence the fact that immigrants no longer choose to migrate to European countries for fear of prejudice and rejection and the increase in the movement to close the doors to refugees^(17)^. The principles which guarantee this access in Brazil are the same principles of the Unified Health System for the entire population: Universality, Comprehensiveness and Equity. However, this population who are characterized by spontaneously seeking services due to structural issues, experiences several difficulties^(18)^.

With regard to the investigated municipal healthcare network to assist black immigrants during the Covid-19 pandemic, it was identified that the organization happened in a general way and not specifically to immigrants infected by the coronavirus. PHC is responsible for notifying, detecting and monitoring positive cases^(19)^. In the HUs participating in the study, organizing the flow of symptomatic and non-symptomatic patients became a preventive measure for the virus spread.

In this context of reorganizing the healthcare network, initiatives were reported to support vulnerable groups that took place through establishing contact with local leaders, equipment and institutions, which were articulated with other community initiatives^(19)^. A well-organized and managed PHC can provide safe conditions for this care, especially for people who are in situations of greater vulnerability^(20)^.

There was a system during the pandemic that despite being organized based on healthcare networks in order to guarantee comprehensive care, neglected race and ethnicity in the surveillance of cases from monitoring the mildest cases to those which resulted in deaths. In addition, health education proposals also moved away from racial issues, which may have been able to increase the historical vulnerabilities in these populations^(8)^.

One study which sought to identify the health condition of Haitians in southern Brazil regarding respiratory symptoms reported that there are high rates of pathologies such as tuberculosis due to precarious living and working conditions, which reduce the immune status compared to that of the local population^(21)^. According to a report by the Institute for Applied Economic Research (*Instituto de Pesquisa Econômica Aplicada - IPEA*), immigrant and refugee populations in Brazil have more cases of HIV/AIDS, tuberculosis, leishmaniasis and malnutrition, constituting situations associated with poor housing and employment conditions^(22)^.

In the speeches of the participants, it was verified that the majority of immigrant women presented needs in gynecological care, prenatal care and continuity in this care for their children. This reality is confirmed by the demonstration of the condition in several studies which point to this practice as being common, especially among Haitian women in different states of Brazil and in European countries^(23)^.

A fact highlighted in this study was the reference to the lack of training in providing care to the black immigrant population and consequently to the specific factors that affect their health, as well as the National Policy for Comprehensive Health of the Black Population. The literature highlights PHC as the gateway to the Brazilian public health system, working directly with the population, and acting as a transformer of social practices. However, for this to happen, these actions need to be mediated by training which generates the professionals’ understanding of the reality in which the population served is inserted, and from there, the need for change^(24)^.

The normalizing discourse when referring to a supposed equity in healthcare between black immigrants and non-immigrants was evidenced in some reports of this study. It is estimated that the use of this term may be associated with an attempt to trivialize the social racial structure linked to social institutions of different segments, including health, which absorb or normalize the action of structural racism in all individuals^(9)^. Body identities exist and bear marks of their classification meanings. Therefore, the discourse that we are all the same or that the care is normal for everyone exposed in the participants’ speeches reveals a sense as if the black body did not exist; however, identification of the black being will always exist, carrying with it all self-representation^(25)^.

The implementation of programs aimed at cultural education and anti-racist training in the reality of health professionals will make it possible to recognize, in health systems and socially, the complexity and damage that racism causes in living conditions, in addition to interfering with the health of the black population that suffers from this discrimination^(26)^. However, actions like these are not easily implemented, as demonstrated by a study carried out with health professionals from Sweden, Germany and Portugal, in which the difficulty in carrying out a theoretical exploration of racism within health spaces was discussed. This theme is little punctuated and conceptualized in healthcare, being scarce in health meetings. Racism is acted out in a subtle, invisible and normalized way in medical routines, which induces the limitation or denial of access to basic human needs and a dignified quality of life^(27)^.

The consequences of structural racism include: childhood trauma, discrimination, health disparities, health inequities, prejudice, racial trauma, social determinants of health, and economic status. Such factors favor the appearance of heart diseases; thus, health professionals should be trained from the beginning of their training to be able to develop a more sensitive look at the race debate^(28)^.

Some speeches pointed out that men have more command of the Portuguese language than women, which may be because of their greater insertion in the labor market due to the opportunities being greater for black immigrant men. The existence of the sexual division at work has consequences on the social organization of immigrants, mainly on the development of language skills^(29)^.

A study carried out in Amazonas with Haitian immigrant women pointed out that they carry identity, social, cultural and historical factors with them, and that they seek immigration as a way to provide for the needs of family members who remained in the country of origin. Despite the difficulties, several managed to enter the job market, as well as achieve reasonable command of the Portuguese language. It was also found that they contribute to household expenses and welcome compatriots into their homes, establishing a support network^(30)^.

A limitation of this study (already mentioned) was the lack of details in the registration of the black immigrant population, making it necessary to resort to NGOs which promote attention to the black immigrant population to know their location. Another limitation was due to the pandemic period in which data collection was conducted which caused interruption in the interviews due to a peak of the disease, which made it impossible to visit all the places that had been approved by the ethics committee.

## CONCLUSION

The study showed that the healthcare of the black immigrant population during the Covid-19 pandemic faced barriers to be overcome in the deconstruction of structural racism among PHC health professionals in the investigated municipality. Even so, the strategies used by the participants for providing healthcare to the black immigrant population proved to be efficient. However, training is still necessary to develop skills, especially on the National Policy for the Comprehensive Health of the Black Population and on understanding the culture of other peoples, which present identification of new horizons for the healthcare of this population.

Structural racism is part of the construction movement of our society and mainly as a structure that maintains the economic system, and it is necessary to recognize its existence in order to create strategies to overcome it and anti-racist practices. As a contribution, it was shown that the health and disease process is also determined by racial issues, since these identities are historical and identical.

The consequences arising from the Covid-19 pandemic go beyond its epidemiological data, as understanding the circumstances of the aggravations comes from observing the functioning of the power structures which regulate the social spheres. Social circumstances such as income, access to education, living and working conditions are responsible for the existing inequalities in health and have become more evident during the health crisis.

This study is presented as a theoretical framework for understanding the effects of the historical process in ordering the conjuncture that leads to the phenomenon of social conditions. Health services focused on containing the virus during the Covid-19 pandemic, but did not include comprehensive healthcare for the black immigrant population, which requires formulating management strategies and mitigation policies that result in more humanized healthcare.
